# Annual Meeting of the Texas State Medical Association

**Published:** 1898-05

**Authors:** 


### Society Notes.

### ANNUAL MEETING OF THE TEXAS STATE MEDICAL ASSOCIATION.

### Held in Houston April 26, 27, 28 and 29, 1897.

The thirtieth annual meeting of the Texas State Medical Association was called to order at 11:45 a. m., April 26th, by Dr. Joseph A. Mullen, chairman of the local arrangement committee, who introduced Rev. H. D. Aves. Rev. Aves, after repeating the Lord's prayer, delivered a fervent invocation.

Mayor Sam H. Brashear welcomed the visitors on the part of the city and Hon. J. W. Jones welcomed them on the part of the citizens of Houston.

Dr. Mullen then announced the social features of the meeting.

President Bacon Saunders of Fort Worth, in responding to the addresses of welcome, said that the Texas State Medical association was more than glad to again meet in Houston, its birthplace thirty years ago. The business of the meeting was somewhat serious, but every one knew that doctors saw enough serious things in their daily life, and they were, therefore, all the more glad to accept the warm-hearted hospitality extended them by the physicians of Houston, and by the citizens through Mayor Brashear and Mr. Jones. In closing he earnestly

---

[For the data of this report we are indebted to the Houston Daily Post, which published the full proceedings from day to day.—Ed.]

thanked the speakers who had preceded him for their hospitable utterances.

The business of the meeting was then taken up. After the calling of the roll by Secretary West, the reading of the minutes of the previous meeting was announced, but on motion this feature was dispensed with.

Secretary West then presented his annual report:

Membership on the roll, transactions, 1897:

| | |
|---|---|
| Honorary | 26 |
| Ordinary | 244 |
| Total | 270 |
| Elected at Paris, ordinary | 19 |

Dropped from the roll since 1896:

| | |
|---|---|
| By death | 5 |
| By non-payment of dues | 62 |
| Total dropped | 67 |

For printing the transactions the bid of the Von Boeckmann Publishing company of Austin, being the lowest, was accepted. The bill is as follows:

Copies, 400; paper, 390; Morocco, 10.

| | |
|---|---|
| 69 pages brevier, $1 per page | $ 69 00 |
| 139 pages Long Primer, at 90c per page | 125 10 |
| Printing and inserting portrait | 4 00 |
| Binding ten copies in Morocco | 7 50 |
| Addressing and mailing | 3 00 |
| Postage | 24 68 |
| Total | $232 28 |

The bill has been paid and I am happy to say a balance left in the treasurer's hands.

Cost per volume delivered about 57 cents.

Dr. J. T. Wilson, of Sherman, moved that the report be received and referred to a committee. The motion prevailed, and President Saunders appointed Dr. W. R. Blailock, of McGregor; Dr. P. C. Coleman, of Colorado City; Dr. J. C. Loggins, of Ennis, to act as such committee.

The annual report of Treasurer J. Larendon was then presented as follows:

TREASURER'S REPORT.

Dr. J. Larendon in account with the Texas State Medical Association.

RECEIPTS.

| | |
|---|---|
| To amount cash balance on hand as per last annual report | $ 80 31 |
| To amount cash collected from members for dues up to date | 1,028 00 |
| Total | $1,108 31 |

DISBURSEMENTS.

| | |
|---|---|
| By cash paid H. A. West, secretary's salary | $ 300 00 |
| By cash paid Frank C. Pierce, stenographer | 50 00 |
| By cash paid State franchise tax | 10 00 |
| By cash paid J. Larendon, treasurer's salary | 75 00 |
| By cash paid U. S. for postage | 10 00 |
| By cash paid Eugene Von Boeckmann on last year's account | 153 90 |
| By cash paid Allen & Co., for gold medal | 15 00 |
| By cash paid J. Riordan, for preparing delinquent list | 1 00 |
| By cash to A. C. Gray, for printing | 5 00 |
| By cash paid U. S. for postage on extra circulars | 5 00 |
| By cash paid Eugene Von Boeckmann for printing transactions | 233 28 |
| By cash paid for exchange on six drafts | 80 |
| By cash balance on hand | 249 33 |
| Total | $1,108 31 |

Respectfully submitted,

J. LARENDON, Treasurer.

On motion the report was referred to the committee named above.

President Saunders next presented his annual message, which was also referred to the committee.

The meeting then adjourned to 2:30 p. m.

### AFTERNOON SESSION.

The afternoon session was called to order at 2:45, at which time the section on general medicine was taken up. Chairman A. B. Gardner being absent, Dr. Loggins presided in his stead.

The first paper read was one on "Differential Diagnosis Between Yellow Fever and Dengue, with Some Account of the Epidemic in Texas of 1897," by Dr. H. A. West, of Galveston; discussed by Dr. George H. Lee, of Galveston, and Dr. R. T. Morris, of Houston.

A paper on "The Decadence of the Drug Treatment of Disease" was next presented by Dr. Frank B. King, of Houston. No discussion further than an expression of approval from Dr. Reeves, of Comanche, followed it.

"A Pathological Problem—Fever, What Is It?" was the next paper, which was presented by Dr. J. M. Fort, of Paris. Dr. W. R. Blailock, of McGregor, opened the discussion, which was not further proceeded with on account of the lateness of the hour.

On motion of Dr. R. H. Harrison, of Columbus, the meeting adjourned to 8 p. m.

### NIGHT SESSION.

The night session was convened at 8 o'clock, at which time the discussion on Dr. Fort's paper was continued. Those who presented their views were Dr. Wilson, of Sherman, and Dr. Frazier, of Belton, Dr. Fort closing.

Dr. Gardner, chairman of the Section, had arrived since the close of the afternoon session, and at this juncture presented his report, which was referred to the Publication Committee.

A paper on "The Prevention of Tuberculosis" was next read by Dr. M. M. Smith, of Austin. Discussed by Drs. S. C. Red, Morgan, Fort, R. W. Knox and H. A. West. Dr. Smith closed.

The hour of 10:30 having arrived, a motion to adjourn to 9 o'clock next morning was made and carried.

### THE STUART RECEPTION.

A reception was given by Dr. and Mrs. D. F. Stuart, at their home, between the hours of 5 and 8 p. m., Tuesday, and was well attended. It was given especially in honor of the wives and daughters of the visiting physicians.

### SECOND DAY'S SESSION.

The most of the morning session was consumed in the discussion of the report of Dr. J. T. Wilson, chairman of the Committee on Legislation. Dr. Blailock, of McGregor, moved to adopt the report, and Dr. Archer moved to amend so as to continue the committee, thank it for its labor and adopt its report as a whole. The motion was unanimously adopted as amended.

A motion to invite all visiting members of the profession who did not belong to the Association to seats on the floor, prevailed unanimously.

The business session was then declared closed by President Saunders, who announced that the section on obstetrics and diseases of children would be taken up. A message having been received from Secretary T. L. Kennedy of this section to the effect that he would not be able to get here until today had been received, however, and the section on surgery was, therefore, taken up and opened by the report of the chairman, Dr. B. E. Hadra of San Antonio.

Section No. 2 was taken up first and a paper on "Fractures and Dislocations of the Astragalus and sub-Astragaloid Dislocations" was read by Dr. J. E. Thompson of Galveston. It was made all the more interesting by the exhibition of a dissected foot, a plaster cast, X-ray pictures of the injuries touched on, and other illustrations.

Chairman Hadra announced that all discussions would be deferred until all papers in the section had been read, and Dr. J. E. Gilcreest of Gainesville presented a well prepared article on "The Fracture of the Tarsal Bones." It developing that none of the authors of the other papers in the section were present, further action in the matter was postponed and President Saunders announced that the report of the committee on officers' reports would be made by Chairman Loggins. It was as follows:

*B. Saunders, M. D., President Texas State Medical Association, Houston.*

Your committee to whom was referred the recommendations of the president bear cheerful testimony to the able and efficient zeal which he has manifested in the discharge of his official duties as president of the Texas State Medical association. As evidence of the fact we refer with pride to the large attendance of the profession and the interest manifested at this meeting. We recommend, as suggested by the president, that the treasurer be instructed to draw by sight draft on members in arrears for their dues not later than July 1 after the meeting of the association at which their dues are due and payable, and if payment is not made by September 1 next, that their name be dropped from the roll for non-payment of dues. We heartily and unconditionally indorse the action of our president in writing in his official capacity as president of the Texas State Medical association to our representatives in congress and the senate of the United States calling their attention to the iniquities of the anti-vivisection bill pending in congress, and urging upon them to use all legitimate means for the defeat of the same. We further indorse the president's recommendation that the association in convention assembled take formal action in regard to this measure, which resolution shall be engrossed and copies forwarded to each representative.

*B. Saunders, President Texas State Medical Association:*

Your committee to whom was referred the annual report of the secretary and treasurer, beg leave to report as follows: An examination of the treasurer's report shows the same to be correct, and we recommend that it be received and adopted. The report of the secretary we find correct and would recommend that it be received and adopted, and that the secretary be instructed in the future, as he suggests, to abstain from putting the name of any member of the profession resident of this State on the programme to read any paper or to participate in discussion who is not a member of the association.

Respectfully,

P. C. Coleman,
W. R. Blailock,
J. C. Loggins.

The question of a State board of health is one of such vital importance that your committee would recommend the appointment of a special committee to report at an hour which may be designated, when this association can give it the serious, thoughtful consideration its supreme importance merits.

Respectfully,

P. C. Coleman,
W. R. Blailock,
J. C. Loggins.

The report was adopted.

Secretary West moved that a special committee be appointed to act on the recommendations contained in the president's mesage in regard to the vivisection bill now before congress. The motion prevailed and President Saunders named as such committee Drs. West, Sears and Wilson.

Dr. Lemond of Denver extended to the association an invitation to attend the meeting of the American Medical Association.

Chairman Mullen of the local arrangement committee made some announcements regarding the barge trip and the meeting then adjourned to 9 p. m.

In the afternoon many of the members accompanied by their wives and friends went on a barge excursion down the bayou and to the San Jacinto battle grounds.

The entire party numbered something like 450 people. The train bearing the excursionists arrived at Houston on the return trip shortly after 10 p. m.

### THE THIRD DAY'S SESSION.

The association meeting was called to order shortly after 9 o'clock by President Bacon Saunders. The president called on Dr. I. M. Cline of Galveston, who gave a verbal report on behalf of the committee on climatic conditions and mineralogical resources of the State.

The section on surgery was again opened and a report of two cases of appendicitis was presented by Dr. Blailock of McGregor. The discussion was opened by Dr. Jameson of Palestine, who was followed by Dr. Jelks of Hot Springs and Dr. Hadra of San Antonio. Dr. Blailock closed.

An interesting report on a gun shot case in which the intestines of the patient were perforated, was read by Dr. A. C. Scott of Temple. In discussing the paper Drs. Blailock and Saunders paid its author some well-deserved compliments.

The section on State medicine was opened by Dr. Blailock in the absence of Chairman S. F. White of Terrell. None of the authors of the papers named in the section were present, however, and the section on gynecology was taken up and opened by Chairman H. K. Leake of Dallas, who read his report.

He was followed by Dr. S. M. Jenkins of Summers Mills, who presented a paper on "Surgical Treatment of Retro-displacements of the Uterus." It was discussed by Drs. Paine of Galveston and Phenix of Alvin.

A paper on "Operation for Removal of Right Tube Ovary and Appendix," written by Dr. B. F. Kingsley of San Antonio, was read by Secretary West. It was not discussed.

Dr. A. C. Scott of Temple next read an able paper on "The Restitution of the Perineal Body," which he illustrated by the use of drawings. Dr. W. T. Brown of Wallis and Dr. Jelks of

Hot Springs, Ark., discussed the paper. The noon hour having arrived the section was temporarily closed.

President Saunders announced as the committee recommended in the report of Dr. Cline, Dr. Seth Morris of Galveston, Dr. T. J. Bennett of Austin and Dr. H. A. West of Galveston.

Dr. Lemond of Denver, an honorary member of the association, asked leave to pay dues. He was ruled out of order.

Dr. Mullen, chairman of the local arrangement committee, extended a general invitation to those present to visit the Houston infirmary. He stated that refreshments would be served to all callers.

President Saunders named Dr. J. T. Wilson of Sherman and Drs. M. M. Smith and S. E. Hudson of Austin as a committee to prepare an address fully explaining the true nature of the proposed medical bill, its scope and possibilities. The address is to be published in circular form. Five hundred copies are to be furnished the legislative committee and the remainder to be turned over to the caucusing committee to be distributed among the members of the profession in the State.

Dr. T. J. Bennett of Austin, Dr. J. C. Anderson of Granger, Dr. P. C. Coleman of Colorado City, Dr. W. R. Blailock of McGregor and Dr. J. M. Frazier of Belton were appointed a committee to canvass the State by correspondence with each physician whose name can be obtained in order to work up interest in the medical bill and carry it to a successful issue.

Dr. J. H. Sears of Waco, one of the oldest members of the association, who, during the morning session had been made an honorary member, arose to protest against such action. He had, he said, been an active member for thirty years and he wished to continue as such until death claimed him. His declaration was loudly applauded, but he was continued on the honorary roll.

After announcing that the representatives of the different congressional districts in the State were requested to get together and appoint a nominating committee. President Saunders adjourned the meeting to 2 p. m.

### AFTERNOON SESSION.

Immediately after the convening of the afternoon session President Saunders announced the following nominating committee, the members having been chosen by the representatives of their respective congressional districts during the noon hours: First district, J. W. Scott of Houston; Second, F. C. Ford of Nacogdoches; Third, J. D. Becton of Greenville; Fourth, J. M. Fort of Paris; Fifth, R. F. Miller of Sherman; Sixth, J. C. Loggins of Ennis; Seventh, A. C. Scott of Temple; Eighth, C. S. Bobo of Weatherford; Ninth, M. M. Smith of Austin; Tenth, I. E. Clark of Schulenberg; Eleventh, T. G. Duncan of Victoria; Twelth, B. E. Hadra of San Antonio; Thirteenth, P. C. Coleman of Colorado City.

A paper from Dr. Thomas Moore Madden of Dublin, Ireland, an honorary member of the association, was received with thanks.

The section on gynecology was resumed and Dr. J. D. Becton of Greenville read a report on a case of vicarious menstruation, which went to the publishing committee without discussion.

The section on ophthalmology and otology was opened by the report of Chairman R. F. Miller of Sherman.

This was followed by a paper on "Early Operation in Pterygium," by Dr. W. R. Thompson of Fort Worth.

"Some Ophthalomological Don'ts" were presented by Dr. S. L. Terrell of Dallas, and the paper was discussed by Drs. Hulen of Galveston and Thompson of Fort Worth.

"The Relation of our Section to the State Medical Association," a very interesting paper, was read by Dr. Vard H. Hulen of Galveston. The discussion was participated in by Drs. Lemond, Anderson, Mullen, Thompson, Miller and Daviss, and closed by the author.

Dr. J. A. Mullen's paper on "Supra-Renal Capsule Extract as an Adjuvant to Cocaine in Minor Operations of the Eye and its Appendages," was discussed by Dr. Boyd of Fort Worth, Dr. Lemond of Denver, Dr. Daviss of Houston, Dr. Wilson of Sherman, Dr. Thompson of Fort Worth and Dr. Keiler of Galveston.

A report of a case of glaucoma, by Dr. R. H. Chilton of Dallas, was read by Dr. Terrell of the same city. It went to the publication committee without comment.

A paper on "Superficial Keratitis," prepared by Dr. F. D. Boyd of Fort Worth, was read by its author. It was briefly discussed by Dr. Thompson.

"The Evolution of the Artificial Pupil" was the subject allotted to Dr. E. P. Daviss of Houston.

Dr. Lemond of Denver read a very interesting account of a case in which he transplanted a rabbit's cornea to the eye of a man. The paper was discussed by Dr. Hulen, Dr. Wilson and Dr. Thompson. Dr. Lemond closing.

Dr. Lemond again requested all those who proposed to attend the American Medical Association to be held at Denver on June 7 to 10, give him their names in order that proper accommodations might be prepared for them.

The section on obstetrics and diseases of children was opened by the report of Secretary T. L. Kennedy of Galveston.

The paper on "Eclampsia-Etiology," by Dr. W. F. Starley, Jr., of Galveston, was laid over for further action.

Dr. T. W. Shearer of Wallisville read an article on the symptoms of eclampsia, which was discussed by Dr. Red of Houston and Dr. Sears of Waco.

The "Treatment of Eclampsia" was handled by Dr. Blailock of McGregor in a very able manner. It was discussed by Dr. Smith of Austin, after which the meeting adjourned to 8 p. m., and most of the members of the association with a number of their lady friends boarded the street cars provided for them and were taken around the principal belt lines of the city.

### NIGHT SESSION.

A crowd which comfortably filled the hall was present at night to listen to the annual address of President Saunders.

This address was delivered in Dr. Saunders' usual happy style and was really a masterful effort. We regret that our space is too limited to print it in full.

### THE FOURTH DAY'S SESSION.

The association met at 9 o'clock. The committee on nominations of officers and on next place of meeting submitted the following report:

Houston, Texas, April 29.—Mr. President and Gentlemen of the Association: Your nominating committee beg leave to report to your honorable body the following nominations: For president, Drs. J. T. Wilson of Sherman and A. B. Gardner of Bellville.

First vice president, Dr. J. M. Fort of Paris; second vice president, Dr. Taylor Hudson of Belton; third vice president, Dr. R. W. Knox of Houston.

Judicial council: Drs. G. T. Thomas of Rogers, H. K. Leake of Dallas, T. G. Duncan of Victoria and A. C. Scott of Temple.

Orator: Dr. M. M. Smith of Austin.

Publishing committee: Drs. J. F. Y. Paine and Vard H. Hulen of Galveston.

Delegates to the American Medical association: Drs. S. C. Red of Houston; J. M. Fort, Paris; J. Kaiser, Flatonio; F. O. Norris, Eagle Lake; P. C. Coleman, Colorado City; J. E. Gilcreest, Gainesville; B. F. Kingsley, San Antonio; S. E. Hudson, Austin; T. J. Wagley, Cleburne; H. A. West, Galveston; J. T. Wilson, Sherman, Bacon Saunders, Fort Worth; Allen J. Smith, Galveston; G. H. Smith, Galveston; J. W. Miller, Hillsboro; and J. F. Y. Paine, Galveston.

It is further moved that the secretary be instructed to issue delegate certificates to any member of this association who attends the American Medical association at Denver, Colo.

Place of meeting for 1899, San Antonio, Tex.; chairman of arrangements, Dr. B. E. Hadra, San Antonio.

Respectfully submitted,

J. C. Loggins, Chairman;
M. M. Smith, Secretary.

The report of the committee was adopted.

Dr. Gardner, who had been recommended for president, asked that the association unanimously elect Dr. Wilson, the other physician recommended for the office, and Dr. Wilson was elected president for the ensuing year.

The secretary and treasurer's term of office do not expire until next year, and therefore no recommendations were made for these offices.

Dr. J. T. Wilson of Sherman was introduced as the next president of the association, and made a happy little talk in accepting the office.

Dr. Bacon Saunders, the retiring president, extended his thanks to the association for their assistance during his term of office.

Dr. Loggins called attention to the fact that the committee to report on a State board of health had not been appointed and the chair appointed Drs. Loggins, Coleman and Scott as this committee.

Dr. Matthew M. Smith of Austin then submitted a paper entitled "Some Remarks on Hypnotism," in which he took the ground that there were some diseases that could not be successfully treated without hypnotism. He thought only physicians should be permitted to use hypnotism and then only in the treatment of diseases. This paper was referred to the publishing committee.

Dr. A. H. Schenk of Kenney read a paper on "The Course and Treatment of Mixœdema with Spontaneous Dislocation of the Hip Joint," which was an exceedingly interesting case. The paper was discussed by Dr. Bacon Saunders, Drs. Harrison and Shearer.

Dr. T. M. Wilson of Thornton then read a paper on "The Treatment of Typhoid Fever."

The paper was discussed by Drs. H. A. West, A. H. Schenk, S. C. Red, Bat Smith, Jelks, Scott and Coleman.

The next paper was on "Hydrotherapy," and was read by Dr. J. H. McCracken, of Mineral Wells.

The committee on a State board of health reported through the chairman, Dr. Loggins, recommending that Drs. R. H. Harrison, M. M. Smith, Bacon Saunders, J. D. Osborn and S. C. Red be appointed a committee to prepare a bill creating a State board of health and that this committee be known as legislative committee No. 2. The report was adopted.

The committee on auxiliary societies reported recommending that no initiation fee or dues be required of county societies, and the report was adopted.

On motion, the association decided to meet next year at San Antonio on the fourth Tuesday in April.

President Wilson, having vacated the chair to Vice President Fort, offered resolutions of thanks to the ladies of Houston, the physicians and the local committee for Houston and the press and the officers of the association.

Dr. Wm. Gammon of Galveston, chairman of the section on microscopy, then read his report on "Bacteria."

The following appointments were made by the vice presidents for the next meeting of the State association:

Section on general medicine: Dr. Geo. H. Lee, Galveston, chairman; Dr. W. F. Starley, Jr., Galveston, secretary.

Section on obstetrics and diseases of children: Dr. C. M. Alexander, Coleman, chairman; Dr. J. M. Frazier, Belton, secretary.

Section on surgery: Dr. A. C. Scott, Temple, chairman; Dr. Marvin Graves, Waco, secretary.

State medicine and public hygiene: Dr. J. C. Erwin, McKinney, chairman; Dr. J. O. McReynolds, Dallas, secretary.

Section on gynecology: Dr. A. N. Denton, Austin, chairman; Dr. S. E. Hudson, Austin, secretary.

Section on ophthalmology: Dr. J. A. Mullen, Houston, chairman; Dr. R. E. Moss, San Antonio, secretary.

Committee on necrology: Dr. I. N. Suttle, Corsicana, chairman; Dr. T. J. Bennett, Austin; Dr. G. T. Thomas, Rogers.

Surgery—Subject: "Intra-Capsular Fractures of the Hip Joint." Dr. R. Nichols, Greenville; discussion by Dr. I. E. Clark, Schulenberg.

State medicine and public hygiene—Subject: "The Water Supply of Texas and Its Relation to the Cause of Diseases." Presentation by Dr. W. M. Terrell, Farmer; discussion by Dr. A. O. Scarborough, Schulenburg.

Gynecology—Subject: "The Proper Supports for the Uterine Displacement." Presentation by Dr. J. W. Scott, Houston; discussion by Dr. F. Herff, Jr., San Antonio.

Ophthalmology—Subject: "The Treatment of Granulated Lids." Presented by Dr. F. D. Boyd, Fort Worth; discussion by Dr. R. F. Miller, Sherman.

PART II—GENERAL MEDICINE.

"La Grippe—Etiology, Pathology, Diagnosis and Treatment"—To present the subject: Dr. J. E. Gilcreest, Gainesville; to open discussion, Dr. P. C. Coleman, Colorado City.

Obstetrics and diseases of children—Subject: "The Prophylactic Treatment of the Pregnant Woman." To present the subject, Dr. J. F. Y. Paine, Galveston; to open discussion, Dr. F. D. Thompson, Fort Worth.

At the close of this feature a very able address was delivered by the orator of the organization, Dr. I. N. Suttle of Corsicana. It was a happy effort; and that it was appreciated was shown by the hearty applause it elicited from the audience.

The business of the session being thus closed, the social feature of the evening was taken up. A program of dances consisting of four waltzes, three two-steps, and two "Lancers" was enjoyed.

### NEW MEMBERS ELECTED.

The only members of the Judicial Council present when the convention met Tuesday morning were Drs. C. S. Bobo, Weatherford; J. D. Osborne, Cleburne; A. B. Gardner, Bellville; and Taylor Hudson, Belton. President Saunders appointed to fill the places of absentees Drs. George H. Lee, Galveston; S. C. Red and W. A. Archer, Houston; P. C. Coleman, Colorado City, and G. T. Thomas, Rogers.

The council met during the noon hour and organized by electing Dr. Bobo chairman and Dr. Lee secretary. During the meeting the following new members were enrolled: Drs. Fred B. Johnson, Grimes county; W. A. Rape, Victoria county; J. A. Youngkin, Lavaca county; C. J. Schraum, Fayette county; W. R. Thompson, Tarrant county; C. B. Phillip, DeWitt county; Max Urwitz, Harris county; J. L. Short, Harris county; Joe Gilbert, Travis county; J. C. Anderson, Williamson county, H. W. Cummings, Robertson county; M. A. Weems, Brazoria county; N. J. Phenix, Brazoria county; F. G. Eidman, Harris county; Donald McKay, Harris county; J. D. Dorbandt, Lampasas county; C. C. Myers, Waller county; W. T. Brown, Austin county; R. R. White, Bell county; R. W. Hix, Wilbarger county; J. B. Massie, Harris county; P. M. Royson, Brazos county; M. B. Hollis, Houston county; J. S. Zvesper, Fayette county; J. W. Scott, Harris county; A. J. Jeter, Ellis county; H. W. Dudley, Hill county; J. R. Reeves, Blanco county; J. J. Roberts, Hill county; R. T. Morris, Harris county; E. E. Grant, Harris county; R. A. Harris, Bandera county; M. L. Graves, McLennan county; J. L. McLeron, Harris county; E. B. Jackson, Harris county; J. H. Logan, Mills county; A. W. Ireland, Washington county; F. W. Kaiser, Fayette county; J. V. Cochran, Limestone county; S. H. Hill, Harris county; G. W. Allen, Fayette county; Frank Karnes, Colorado county; E. M. Shaw, Victoria county; K. N. Miller, Austin county; E. A. Thompson, Grimes county; C. E. Gwinn, Cherokee connty; J. M. Mason, Wilson county; E. L. Batts, Galveston county; W. B. Pullen, Nacogdoches county; M. C. Bell, Austin county; J. C. Mayfield, Galveston county; H. H. McConnelly, Wharton county; M. Grumley, Harris county; D. M. Thurston, Bee county; C. Scovarizo, Brazoria county; Minnie C. Archer and O. L. Norsworthy, Harris county; J. A. Gibson and Bat Smith, Wharton county; B. F. Calhoun, Jefferson county; T. R. Ogden.

The meeting was a most successful one in many respects. The attendance was larger than for many years past, and the scientific papers read and discussed at this meeting will compare favorably with any similar meeting or medical body in the country. The programme was carried out almost in full, and the social features of the meeting were many and were much enjoyed by the visitors. The profession and citizens of Houston left nothing undone to insure the comfort and pleasure of the visiting physicians and their wives, who accompanied them, and the Houston meeting will be long and pleasantly remembered by many of our hard-worked physicians.

The association adjourned to meet in San Antonio on the fourth Tuesday in April, 1899.

